# Supplementary material for: Hot spot prediction in protein-protein interactions by an ensemble system
Source: BMC Syst Biol. 2018 Dec 31;12(Suppl 9):132. doi: 10.1186/s12918-018-0665-8 (PMC6311905; doi:10.1186/s12918-018-0665-8)
Supplement: Supplementary file 2 — All relevant MATLAB code. A simple Matlab implement of our predictor. (ZIP 19,742 kb) [file 12918_2018_665_MOESM2_ESM.zip › Additional file 2/Read ASA/ASA Property/ASEdb/1BRSD.txt]

 NetSurfP - Protein Surface Accessibility and Secondary Structure Predictions

  

|  |  |  |
| --- | --- | --- |
|  |  | NetSurfP - Protein Surface Accessibility and Secondary Structure Predictions  Technical University of Denmark |

  


---

```
# For publication of results, please cite:
# A generic method for assignment of reliability scores applied to solvent accessibility predictions.
# Bent Petersen, Thomas Nordahl Petersen, Pernille Andersen, Morten Nielsen and Claus Lundegaard
# BMC Structural Biology 2009, 9:51 doi:10.1186/1472-6807-9-51
#
# Column 1: Class assignment - B for buried or E for Exposed - Threshold: 25% exposure, but not based on RSA
# Column 2: Amino acid
# Column 3: Sequence name
# Column 4: Amino acid number
# Column 5: Relative Surface Accessibility - RSA
# Column 6: Absolute Surface Accessibility
# Column 7: Z-fit score for RSA prediction
# Column 8: Probability for Alpha-Helix
# Column 9: Probability for Beta-strand
# Column 10: Probability for Coil
E K  1BRSD                  1    0.748 153.864   1.611   0.005   0.015   0.979
E K  1BRSD                  2    0.444  91.310   2.099   0.001   0.900   0.099
B A  1BRSD                  3    0.071   7.868   0.690   0.001   0.959   0.040
E V  1BRSD                  4    0.329  50.521   1.242   0.000   0.983   0.017
B I  1BRSD                  5    0.059  10.823   0.233   0.001   0.959   0.040
E N  1BRSD                  6    0.297  43.452   0.525   0.001   0.900   0.099
B G  1BRSD                  7    0.100   7.909  -1.289   0.021   0.279   0.699
E E  1BRSD                  8    0.535  93.377   0.702   0.018   0.088   0.893
E Q  1BRSD                  9    0.499  89.193   1.645   0.020   0.205   0.775
B I  1BRSD                 10    0.042   7.714   0.181   0.019   0.141   0.840
E R  1BRSD                 11    0.483 110.630   0.880   0.018   0.088   0.893
E S  1BRSD                 12    0.532  62.304   0.759   0.018   0.019   0.964
E I  1BRSD                 13    0.227  42.069   1.648   0.923   0.002   0.076
E S  1BRSD                 14    0.659  77.211   1.072   0.923   0.002   0.076
E D  1BRSD                 15    0.469  67.597   1.898   0.970   0.001   0.030
B L  1BRSD                 16    0.047   8.679   0.909   0.970   0.001   0.030
B H  1BRSD                 17    0.140  25.539   1.232   0.970   0.001   0.030
E Q  1BRSD                 18    0.577 103.142   1.673   0.970   0.001   0.030
E T  1BRSD                 19    0.311  43.177   1.822   0.988   0.000   0.012
B L  1BRSD                 20    0.043   7.873   0.403   0.970   0.001   0.030
E K  1BRSD                 21    0.242  49.821   1.008   0.970   0.001   0.030
E K  1BRSD                 22    0.681 140.041   1.109   0.923   0.002   0.076
E E  1BRSD                 23    0.369  64.394   0.963   0.782   0.003   0.216
B L  1BRSD                 24    0.102  18.749   0.244   0.406   0.004   0.590
E A  1BRSD                 25    0.708  78.011   0.310   0.018   0.019   0.964
B L  1BRSD                 26    0.170  31.035   0.356   0.003   0.003   0.994
E P  1BRSD                 27    0.602  85.481   0.273   0.016   0.005   0.979
E E  1BRSD                 28    0.703 122.884   0.037   0.053   0.005   0.942
B Y  1BRSD                 29    0.230  49.172  -0.046   0.257   0.016   0.727
B Y  1BRSD                 30    0.213  45.539   0.094   0.430   0.016   0.555
B G  1BRSD                 31    0.269  21.170  -1.195   0.339   0.016   0.645
E E  1BRSD                 32    0.411  71.837   0.254   0.257   0.016   0.727
B N  1BRSD                 33    0.129  18.959  -0.663   0.181   0.016   0.803
B L  1BRSD                 34    0.074  13.531   1.214   0.622   0.015   0.363
B D  1BRSD                 35    0.195  28.128   0.056   0.802   0.014   0.185
B A  1BRSD                 36    0.058   6.337   0.727   0.879   0.010   0.111
B L  1BRSD                 37    0.025   4.486   0.354   0.938   0.007   0.055
B W  1BRSD                 38    0.117  28.042   0.628   0.938   0.007   0.055
B D  1BRSD                 39    0.127  18.301  -0.342   0.879   0.010   0.111
B A  1BRSD                 40    0.040   4.419   0.214   0.879   0.010   0.111
B L  1BRSD                 41    0.045   8.166   0.281   0.717   0.014   0.269
B T  1BRSD                 42    0.274  38.045   0.462   0.430   0.016   0.555
E G  1BRSD                 43    0.462  36.328  -1.663   0.109   0.005   0.886
E W  1BRSD                 44    0.385  92.520  -1.290   0.053   0.005   0.942
B V  1BRSD                 45    0.134  20.627  -1.127   0.058   0.017   0.925
E E  1BRSD                 46    0.632 110.393  -0.648   0.018   0.019   0.964
E Y  1BRSD                 47    0.320  68.320   0.829   0.018   0.019   0.964
E P  1BRSD                 48    0.278  39.448  -0.570   0.018   0.088   0.893
B L  1BRSD                 49    0.118  21.624   1.064   0.003   0.718   0.279
E V  1BRSD                 50    0.303  46.510   1.315   0.001   0.959   0.040
B L  1BRSD                 51    0.041   7.507   0.716   0.001   0.959   0.040
E E  1BRSD                 52    0.209  36.495   1.521   0.001   0.959   0.040
B W  1BRSD                 53    0.059  14.214   0.976   0.001   0.959   0.040
E R  1BRSD                 54    0.426  97.623   1.738   0.004   0.616   0.381
E Q  1BRSD                 55    0.385  68.779  -0.715   0.004   0.138   0.858
B F  1BRSD                 56    0.144  28.961   0.360   0.181   0.016   0.803
E E  1BRSD                 57    0.669 116.857   1.268   0.694   0.003   0.303
E Q  1BRSD                 58    0.518  92.568   1.652   0.782   0.003   0.216
B S  1BRSD                 59    0.061   7.173  -0.024   0.694   0.003   0.303
E K  1BRSD                 60    0.369  75.842   0.605   0.600   0.003   0.397
E Q  1BRSD                 61    0.708 126.449   0.877   0.502   0.002   0.495
E L  1BRSD                 62    0.370  67.802   0.065   0.406   0.004   0.590
B T  1BRSD                 63    0.127  17.670  -0.015   0.176   0.004   0.820
E E  1BRSD                 64    0.456  79.751   0.439   0.694   0.003   0.303
E N  1BRSD                 65    0.669  97.986   1.179   0.858   0.002   0.139
B G  1BRSD                 66    0.137  10.774   0.992   0.923   0.002   0.076
B A  1BRSD                 67    0.053   5.885   0.528   0.970   0.001   0.030
E E  1BRSD                 68    0.520  90.827   1.349   0.970   0.001   0.030
E S  1BRSD                 69    0.267  31.304   2.223   0.970   0.001   0.030
B V  1BRSD                 70    0.015   2.275   1.767   0.970   0.001   0.030
B L  1BRSD                 71    0.161  29.552   1.575   0.970   0.001   0.030
E Q  1BRSD                 72    0.505  90.247   1.964   0.970   0.001   0.030
B V  1BRSD                 73    0.045   6.870   1.047   0.970   0.001   0.030
B F  1BRSD                 74    0.037   7.346   0.352   0.970   0.001   0.030
E R  1BRSD                 75    0.418  95.676   1.675   0.970   0.001   0.030
E E  1BRSD                 76    0.446  77.986   1.619   0.970   0.001   0.030
B A  1BRSD                 77    0.043   4.706   0.223   0.970   0.001   0.030
B K  1BRSD                 78    0.269  55.416   0.622   0.923   0.002   0.076
E A  1BRSD                 79    0.742  81.735   1.100   0.858   0.002   0.139
E E  1BRSD                 80    0.462  80.624   0.316   0.782   0.003   0.216
B G  1BRSD                 81    0.235  18.502   0.465   0.246   0.004   0.750
E A  1BRSD                 82    0.719  79.212   0.326   0.016   0.005   0.979
E D  1BRSD                 83    0.765 110.237   1.001   0.005   0.015   0.979
B I  1BRSD                 84    0.090  16.595   1.115   0.004   0.197   0.799
E T  1BRSD                 85    0.479  66.479   1.336   0.003   0.718   0.279
B I  1BRSD                 86    0.113  20.923   0.504   0.002   0.816   0.182
E I  1BRSD                 87    0.227  41.958  -0.037   0.021   0.756   0.223
B L  1BRSD                 88    0.216  39.495  -0.098   0.004   0.514   0.481
E S  1BRSD                 89    0.748  87.607   0.129   0.003   0.003   0.994

Explain the output.  Go back.
```

---
